# Be Systematic

**Published:** 1855-10

**Authors:** 


### BE SYSTEMATIC.

It will add more to your convenience and comfort through life than you can now imagine. It saves time, saves temper, saves patience, and saves money. For a while it may be a little troublesome, but you will soon find that it is easier to do right than wrong; that it is easier to act by rule than without one.

*Be systematic in everything;* let it extend to the most minute trifles, it is not beneath you. Whitfield could not go to sleep at night, if, after retiring, he remembered that his gloves and riding whip were not in their usual place, where he could lay his hand on them in the dark, on any emergency; and such are the men who leave their mark for good on the world's history. It was by his systematic habits from youth to age that Noah Webster was enabled to leave to the world his great dictionary. "Method was the presiding principle of his life," writes his biographer.

Systematic men are the only reliable men; they are the men who comply with their engagements. They are minute men. The man who has nothing to do, is the man who does nothing. The man of system is soon known to do all that he engages to do; to do it well and do it at the time promised; consequently he has his hands full. When I want any mechanical job done, I go to the man whom I always find busy, and I do not fail to find him the man to do that job promptly, and to the hour.

And more, teach your children to be systematic. Begin with your daughters at five years of age; give them a drawer or two for their clothing; make it a point to go to that drawer any hour of the day and night; and if each article is not properly arranged, give quiet and rational admonition; if arranged well, give affectionate praise and encouragement. Remember that children, as well as grown people, will do more to retain a name, than to make one.

As soon as practicable, let your child have a room which shall be its own, and treat that room as you did the drawer; thus you will plant and cultivate a habit of systematic action, which will bless that child while young, increase the blessing when the child becomes a parent, and extend its pleasurable influences to the close of life. A single unsystematic person in a house, is a curse to any family. A wife who has her whole establishment so arranged, from cellar to attic, that she knows, on any emergency, where to go for a required article, is a treasure to any man, (my experience, reader!) while one who never knows where anything is, and when it is by accident found, is almost sure to find it crumpled, soiled, out of order, such a wife as this latter is unworthy of the name, and is a living reproach to the mother who bore her.

